# Examining the Relationship Between Anxiety, Depression, and Gastrointestinal Symptoms Among University Students: A Campus-Wide Survey Analysis

**DOI:** 10.7759/cureus.69270

**Published:** 2024-09-12

**Authors:** Turki M Alanzi, Mohammed Almumen, Malak Almogrin, Ali Asiri, Raneem Alhalal, Zahra Almuslem, Mazen Alharbi, Maha Alshammari, Jafar Altammar, Ahmed Almarhoun, Lujain A Aldarsi, Hanan Abuseer, Mrooj Almuwallad, Firdevs Isa, Bandar Altuwaylie

**Affiliations:** 1 Health Information Management and Technology, Imam Abdulrahman Bin Faisal University, Dammam, SAU; 2 Internal Medicine, Jubail General Hospital, Jubail, SAU; 3 Laboratory, Imam Abdulrahman Bin Faisal Hospital, Dammam, SAU; 4 Psychiatry, Abha Psychiatric Hospital, Abha, SAU; 5 Medicine, Vision Colleges, Riyadh, SAU; 6 Medicine, Al-Rayan Medical College, Medina, SAU; 7 College of Medicine, Imam Abdulrahman Bin Faisal University, Dammam, SAU; 8 Family Medicine, Ministry of Health, Dammam, SAU; 9 General Practice/Psychiatry, Al-Amal Mental Health Hospital, Ministry of Health, Medina, SAU; 10 Psychiatry, Al-Amal Mental Health Hospital, Ministry of Health, Medina, SAU; 11 Clinical Pharmacy, Ibn Sina National College for Medical Studies, Jeddah, SAU; 12 Medicine, Umm Al-Qura University, Makkah, SAU; 13 College of Pharmacy, University of Hafr Al-Batin, Hafr Al-Batin, SAU

**Keywords:** mental health, university students, gastrointestinal symptoms, depression, anxiety

## Abstract

Study purpose: This study investigates the relationship between anxiety, depression, and gastrointestinal (GI) symptoms among university students through a comprehensive campus-wide survey.

Methods: A cross-sectional online survey design was adopted in this study. A total of 437 students from three public medical universities in Saudi Arabia participated in the survey, which assessed anxiety and depression using standardized scales and GI symptoms through a tailored questionnaire.

Results: The findings reveal a significant overlap between mental health disorders and GI symptoms, highlighting that students experiencing high levels of anxiety and depression frequently report GI issues. This correlation underscores the importance of integrated health strategies to address both mental and physical well-being.

Conclusion: This study concludes that there is a significant correlation between anxiety, depression, and GI symptoms among university students. This highlights the need for comprehensive and integrated health interventions that address both mental and physical health to improve overall well-being and academic success in this population.

## Introduction

Anxiety is a mental health condition characterized by excessive worry, fear, or nervousness about everyday situations. It involves a persistent feeling of apprehension or dread, which can interfere with daily activities. Anxiety disorders encompass various conditions, including generalized anxiety disorder (GAD), panic disorder, social anxiety disorder, and specific phobias [1,2]. Common physical manifestations include increased heart rate, rapid breathing (hyperventilation), sweating, trembling, fatigue, and gastrointestinal (GI) problems such as stomach aches or irritable bowel syndrome (IBS). Individuals with anxiety may experience difficulties in concentrating, racing thoughts, and an overwhelming sense of impending doom or panic [3,4].

Depression is a common and serious mood disorder characterized by persistent feelings of sadness, hopelessness, and a lack of interest or pleasure in activities once enjoyed. It affects how a person feels, thinks, and handles daily activities, including sleeping, eating, or working [5,6]. Emotional symptoms include feelings of sadness, tearfulness, emptiness, or hopelessness, angry outbursts, irritability or frustration, and loss of interest in most or all normal activities. Physical symptoms include unexplained physical problems, such as back pain or headaches, changes in appetite leading to weight loss or gain, and disrupted sleep patterns (insomnia or oversleeping). Individuals with depression may experience difficulty in thinking, concentrating, making decisions, and remembering things, as well as recurring thoughts of death or suicide [7-9].

Anxiety and depression are prevalent mental health issues that significantly affect individuals' overall well-being. Among university students, the pressures of academic performance, social dynamics, and transitioning to adulthood exacerbate these conditions, leading to an alarming increase in their incidence. Concurrently, GI symptoms, such as IBS, constipation, and dyspepsia, are common among this demographic, often reported without a clear organic cause [10-14]. Despite the apparent co-occurrence of anxiety, depression, and GI symptoms, the intricate relationship between these factors remains underexplored within the university student population [15,16].

Existing literature predominantly focuses on either mental health or GI health in isolation. While some studies acknowledge the psychosomatic interplay between mental health disorders and GI symptoms, comprehensive analyses integrating both aspects are scarce [11-14], particularly within the context of university students. This gap highlights a critical need for targeted research to elucidate the interconnectedness of anxiety, depression, and GI symptoms, providing insights that could inform holistic health interventions and support services tailored for students.

The significance of this research lies in its potential to bridge the knowledge gap by offering a nuanced understanding of the psychosomatic dynamics affecting university students. Unveiling the links between mental health and GI symptoms can facilitate the development of integrated health strategies, fostering a supportive campus environment conducive to both mental and physical well-being. Moreover, identifying specific factors that contribute to the co-occurrence of these conditions can aid in early detection and intervention, ultimately enhancing the quality of life and academic performance of students.

The aim of this study is to examine the relationship between anxiety, depression, and GI symptoms among university students through a comprehensive campus-wide survey analysis. The objectives of the research include: i) assessing the prevalence of anxiety, depression, and GI symptoms among university students; ii) identifying potential demographic factors that may influence the relationship between mental health and GI symptoms; iii) analyzing the correlation between anxiety and depression with GI symptoms in this population; iv) providing recommendations for university health services to address the co-occurrence of these conditions effectively.

Literature review

The intersection of mental health issues such as anxiety and depression with GI symptoms is an emerging field of interest in psychosomatic medicine. Understanding how these conditions interrelate, particularly in university students, can offer insights into more effective treatment and support strategies. This literature review examines existing studies on the scales used to measure anxiety, depression, and GI symptoms, highlighting the prevalence, symptomatology, and interrelations among these conditions.

Anxiety scale

Several studies have explored the various dimensions of anxiety symptoms among university students, utilizing different scales to assess their prevalence and impact. Dryness of the mouth, a common physical symptom of anxiety, has been frequently reported by students experiencing high anxiety levels, linking it to the body's stress response mechanisms as noted in recent studies [17,18]. Breathing difficulties, including hyperventilation and breathlessness, are well documented in individuals with anxiety disorders [18]. Leyro et al. [19] highlighted a significant correlation between these breathing issues and panic attacks among university students, emphasizing the physiological impact of anxiety.

Trembling, particularly in the hands, is another physical manifestation that interferes with daily activities and academic performance. It was observed that students with high anxiety scores often reported this symptom [20]. Moreover, the worry about situations in which one might panic and feel embarrassed is a central component of anxiety. Jefferson [21] found this concern prevalent among individuals with social anxiety disorder, significantly affecting their social interactions and participation in academic activities.

Feeling close to panic is a critical symptom in anxiety assessments, with a recent study [22] highlighting that students frequently experiencing this sensation also reported higher overall anxiety levels and lower academic performance. Awareness of heart action, such as experiencing palpitations without physical exertion, is commonly reported by anxious individuals. Another study [23] found a strong association between this symptom and GAD in their study of university students. Lastly, feeling scared without any apparent reason is a hallmark of anxiety. A recent study [24] revealed that this irrational fear significantly impacts students' mental health, leading to avoidance behaviors and increased academic stress.

Depression scale

Depression among university students is characterized by a range of symptoms that severely impact their daily lives and academic success. An inability to experience any positive feelings, known as anhedonia, is prevalent among individuals with high depression scores [25]. This lack of positive affects their social relationships and overall motivation. A lack of initiative to engage in activities is another common symptom. It was observed that depressed students frequently reported difficulties in initiating tasks, contributing to academic underperformance [26].

Feelings of hopelessness and a lack of prospects for the future are central to depression. Several studies [27,28] highlight that these feelings are common among depressed students, leading to a pervasive sense of despair and decreased academic engagement. Similarly, feeling downhearted and blue is a typical emotional state for those with depression. It was noted that this persistent sadness significantly impacts students' daily functioning and overall mental health [29].

An inability to become enthusiastic about anything is another characteristic of depression. This symptom was extensively documented in the study by Mahmoudian [30], who found that students with depression showed a marked lack of interest in previously enjoyed activities. Feelings of worthlessness and the belief that life is meaningless are profound aspects of depression. Studies [31,32] emphasized that these feelings lead to decreased self-esteem and increased risk of suicidal ideation among university students.

GI symptom assessment

GI symptoms are often reported alongside mental health issues among university students. Abdominal pain or discomfort, frequent diarrhea or loose stools, and frequent constipation or difficulty passing stools are common symptoms associated with both anxiety and depression. A study by Hu et al. [33] indicated a significant overlap between these GI symptoms and mental health conditions, suggesting a psychosomatic link.

Bloating or a feeling of fullness after eating, excessive belching or flatulence, and heartburn or acid reflux are also prevalent among students with high levels of anxiety and depression. These symptoms were extensively studied in the study by Ilchmann-Diounou and Menard [34], who found that stress and emotional distress exacerbate these GI issues. Nausea or vomiting, noticing blood in the stool, and experiencing difficulty swallowing or a feeling of food getting stuck are severe symptoms that require medical attention. It was noted that these symptoms often correlate with high stress and anxiety levels, indicating a need for integrated care approaches [35]. Unexplained weight loss is another critical GI symptom that can be associated with severe anxiety and depression. This symptom was highlighted in studies [36,37], which found that students experiencing significant mental health issues often had disordered eating patterns and unexplained weight changes.

The analysis of above findings from the literature review underscores the complex interplay between anxiety, depression, and GI symptoms among university students. The prevalence of these conditions and their interrelated nature highlight the need for comprehensive and integrated health strategies to support students' mental and physical well-being. Further research is essential to develop targeted interventions that address the holistic needs of this population, ultimately enhancing their quality of life and academic performance.

## Materials and methods

A cross-sectional survey design was adopted in this study. The details of the study design are presented in the following sections.

Study setting and participants

Given the context of this study which focuses on examining the relationship between anxiety, depression, and GI symptoms, only university students from different academic courses were considered for this study. Three public medical universities in Saudi Arabia were selected for the study. The inclusion criteria required participants to be 18 years of age or older, currently enrolled as students at one of the selected public medical universities in Saudi Arabia, and from various academic courses within those institutions. This approach ensures a diverse representation of the university student population and captures a range of experiences related to anxiety, depression, and GI symptoms across different fields of study. Conversely, specific exclusion criteria were also established to maintain the integrity of the study. Individuals who are not currently enrolled as students at the selected universities, those under the age of 18, and students with pre-existing medical conditions that could significantly affect GI symptoms, such as diagnosed GI diseases were excluded. Students were recruited through university portals through emails by providing the survey link and the information sheet, explaining the purpose of the study and their rights to participation.

Sampling

Researchers needed an accessible sample since they needed to survey only university students. This study followed the trend of other studies by using a combination of convenience and purposeful sampling [38]. The sample size estimation was performed using Cochran's formula [39], yielding a value of 384.

The sample size estimation was performed using Cochran's formula, which is used to calculate an appropriate sample size for surveys based on desired precision and confidence levels. The formula is as follows:

Where

n0 = required sample size

Z = Z-value (1.96 for 95% confidence level)

p = estimated proportion of the population (we used 0.5, as it provides the maximum sample size)

e = desired level of precision (margin of error), set at 5% (0.05)

Given the finite population correction for the actual number of students available in the selected universities, the adjusted sample size was slightly higher. To ensure adequate power and account for potential non-response, the target sample size was set at 500. The subsequent post hoc power analysis indicated a power of 100%, confirming the adequacy of the sample size for detecting meaningful effects.

Questionnaire design

The questionnaire was designed to examine the relationship between anxiety, depression, and GI symptoms among university students. The study is part of research conducted at Imam Abdulrahman Bin Faisal University in Saudi Arabia, focusing on the relationship between anxiety, depression, and GI symptoms among university students. The survey begins with a consent form where participants agree to take part in the study. This is followed by the first section, which gathers demographic information, including age, gender, and educational level. This section helps to contextualize the findings and allows for subgroup analyses based on demographic variables. The core of the survey consists of three main scales: the Anxiety Scale, the Depression Scale, and the Gastrointestinal Symptoms Assessment. Participants are asked to rate the frequency of their experiences on a 5-point Likert scale, ranging from 1 (Never) to 5 (Always) in all instruments.

Anxiety Scale

This section includes seven items adopted from [40] assessing common anxiety symptoms such as dryness of the mouth, breathing difficulty, trembling, worry about panic situations, feeling close to panic, awareness of heart action, and irrational fears. These items are designed to capture both the physical and psychological manifestations of anxiety.

Depression Scale

This section also consists of seven items adopted from [40], focusing on symptoms of depression. These include the inability to experience positive feelings, lack of initiative, hopelessness, feeling down-hearted, lack of enthusiasm, feelings of worthlessness, and life feeling meaningless. These items aim to cover the emotional and motivational aspects of depression.

Gastrointestinal Symptom Assessment

This section includes 10 items adopted from [33-37] related to GI symptoms. These encompass a range of issues such as abdominal pain, diarrhea, constipation, bloating, belching, heartburn, nausea, blood in stool, difficulty swallowing, and unexplained weight loss. These symptoms are assessed to explore their potential links to anxiety and depression. 

A pilot study was undertaken with a sample of 12 students, and subsequent analysis was performed on the collected data. The Cronbach alpha coefficient was computed for all items and found to exceed 0.7 suggesting good internal consistency and reliability [41].

Data collection

In order to collect information, a questionnaire survey was designed with Google Forms, and it was distributed online through emails and social media applications to the students. At the end of four weeks, a total of 486 responses were received. Out of the 486 responses, 49 students did not fully complete the survey. Therefore, a final sample of 437 was considered for data analysis.

Data analysis

To attain the objectives of the research, the researcher utilized IBM SPSS Statistics for Windows, Version 24 (Released 2016; IBM Corp., Armonk, New York, United States) for analyzing the data. Descriptive statistics were used to characterize the participants’ demographic data. In addition, two-sample t-test with unequal variances and single-factor ANOVA were used for analyzing the data.

Ethics-related factors

The study was approved by the research ethics committee at Imam Abdulrahman Bin Faisal University. All participants provided informed consent before participating. Measures were taken to ensure participant confidentiality and data security, including anonymizing responses and storing data on secure servers. The study adhered to all relevant ethical norms, and no conflicts of interest or funding sources were reported, maintaining research integrity and mitigating bias.

## Results

Based on the demographic data of the participants in the campus-wide survey in Table 1, the majority of students are in the 18-20 and 21-23 age groups, making up nearly half of the respondents (24.94% and 24.49%, respectively). The 24-26 age group constitutes 18.99%, while those aged 27-29 and over 29 represent 14.87% and 16.70%, respectively. Gender distribution shows a predominance of male students at 68.42%, compared to 31.58% female students. Educational background reveals that nearly half of the participants are pursuing a diploma (46.68%), followed by graduates at 31.81% and post-graduates at 21.51%. This diverse demographic profile provides a comprehensive basis for examining the relationship between anxiety, depression, and GI symptoms among university students.

**Table 1:** Participants' demographics

Variable	Groups	N	Relative frequency
Age (in years)	18-20	109	24.94%
	21-23	107	24.49%
	24-26	83	18.99%
	27-29	65	14.87%
	>29	73	16.70%
Gender	Male	299	68.42%
	Female	138	31.58%
Education	Diploma	204	46.68%
	Graduate	139	31.81%
	Post-graduate	94	21.51%

The anxiety scale assessment (see Table 2) among university students reveals varying levels of anxiety symptoms. Dryness of mouth was the most prevalent symptom, with a mean score of 3.28 (SD = 1.09), indicating frequent occurrence among many students. Breathing difficulty (mean = 2.98, SD = 1.45) and trembling (mean = 2.94, SD = 1.13) were also common, though experiences of breathing difficulty showed higher variability. Worrying about panic (mean = 2.58, SD = 1.41) and feeling close to panic (mean = 3.20, SD = 1.87) were moderately common, with the latter showing significant variability. Awareness of heart actions (mean = 2.65, SD = 1.12) and feeling scared without reason (mean = 2.68, SD = 1.39) were similarly moderate in occurrence. These results highlight a range of anxiety symptoms experienced by students, with some symptoms being more prevalent and variable than others.

**Table 2:** Anxiety scale assessment SD: Standard deviation

Items	Mean score	SD
I was aware of dryness of my mouth	3.28	1.09
I experienced breathing difficulty (e.g. excessively rapid breathing, breathlessness in the absence of physical exertion)	2.98	1.45
I experienced trembling (e.g. in the hands)	2.94	1.13
I was worried about situations in which I might panic and make a fool of myself	2.58	1.41
I felt I was close to panic	3.20	1.87
I was aware of the action of my heart in the absence of physical exertion (e.g. sense of heart rate increase, heart missing a beat)	2.65	1.12
I felt scared without any good reason	2.68	1.39

The depression scale assessment (see Table 3) highlights the prevalence of various depressive symptoms among university students. The inability to experience positive feelings had a mean score of 2.78 (SD = 1.57), indicating a moderate frequency of this symptom. Students also found it challenging to initiate activities (mean = 2.73, SD = 1.57) and felt they had nothing to look forward to (mean = 2.56, SD = 1.48). Feeling downhearted and blue was slightly less common (mean = 2.63, SD = 1.41). A notable symptom was the inability to become enthusiastic about anything, with a mean score of 2.81 (SD = 1.56). The feeling of worthlessness was prevalent, with a higher mean score of 2.99 (SD = 1.70), showing significant variability among students. Lastly, feeling that life was meaningless had a mean score of 2.75 (SD = 1.49). These findings suggest that depressive symptoms are moderately common among students, with feelings of worthlessness being particularly significant.

**Table 3:** Depression scale assessment SD: Standard deviation

Items	Mean score	SD
I could not seem to experience any positive feeling at all	2.78	1.57
I found it difficult to work up the initiative to do things	2.73	1.57
I felt that I had nothing to look forward to	2.56	1.48
I felt down-hearted and blue	2.63	1.41
I was unable to become enthusiastic about anything	2.81	1.56
I felt I wasn’t worth much as a person	2.99	1.70
I felt that life was meaningless	2.75	1.49

The gastrointestinal symptoms assessment (see Table 4) among university students indicates varying prevalence and severity of different symptoms. Abdominal pain or discomfort had a mean score of 3.02 (SD = 1.00), making it a common symptom. Frequent diarrhea or loose stools had a slightly higher mean score of 3.09 (SD = 1.12), suggesting it is also prevalent. Constipation or difficulty passing stools was less common (mean = 2.56, SD = 1.65) but showed high variability. Bloating or feeling of fullness after eating (mean = 2.70, SD = 1.69) and excessive belching or flatulence (mean = 2.63, SD = 1.65) were moderately common. Heartburn or acid reflux had a mean score of 2.60 (SD = 1.72), while nausea or vomiting was less frequent (mean = 2.45, SD = 1.62). Noticing blood in stool (mean = 2.48, SD = 1.73) and experiencing difficulty swallowing or a feeling of food getting stuck (mean = 3.06, SD = 1.35) were significant concerns. Unexplained weight loss had a mean score of 2.98 (SD = 1.41). These results indicate that GI symptoms are fairly common among students, with diarrhea, abdominal pain, and difficulty swallowing being particularly notable.

**Table 4:** Gastrointestinal symptom assessment SD: Standard deviation

Items	Mean score	SD
I experience abdominal pain or discomfort.	3.02	1.00
I have frequent diarrhea or loose stools.	3.09	1.12
I have frequent constipation or difficulty passing stools.	2.56	1.65
I experience bloating or feeling of fullness after eating.	2.70	1.69
I have excessive belching or flatulence.	2.63	1.65
I have heartburn or acid reflux.	2.60	1.72
I experience nausea or vomiting.	2.45	1.62
I notice blood in my stool.	2.48	1.73
I experience difficulty swallowing or a feeling of food getting stuck.	3.06	1.35
I have unexplained weight loss.	2.98	1.41

The ANOVA results (see Table 5) assessing the differences in perceptions of anxiety, depression, and GI symptoms among university students highlight several significant findings. Anxiety levels vary significantly by gender and education, but not by age. Females report significantly higher anxiety (mean = 4.16, variance = 0.29) than males (mean = 2.32, variance = 0.29), with a p-value of < .0001. Anxiety also varies by the education level, with diploma holders experiencing the highest anxiety (mean = 3.08, variance = 1.20), followed by post-graduates (mean = 2.89, variance = 0.93) and graduates (mean = 2.65, variance = 0.73), with a p-value of .0005.

**Table 5:** ANOVA results assessing differences between participants' perceptions of anxiety, depression, and gastrointestinal symptoms * Statistically significant difference at p < .05

Factors	Variables		Mean score	Variance	p-value
Anxiety	Age (in years)	18-20	2.74	1.08	.2215
		21-23	2.95	1.00	
		24-26	3.08	1.18	
		27-29	2.87	0.90	
		>29	2.91	0.89	
	Gender	Male	2.32	0.29	< .0001*
		Female	4.16	0.29	
	Education	Diploma	3.08	1.20	.0005*
		Graduate	2.65	0.73	
		Post-graduate	2.89	0.93	
Depression	Age (in years)	18-20	2.58	1.54	.1939
		21-23	2.82	1.58	
		24-26	2.98	1.54	
		27-29	2.79	1.26	
		>29	2.63	1.59	
	Gender	Male	2.15	0.89	< .0001*
		Female	4.06	0.39	
	Education	Diploma	2.88	1.76	.0276*
		Graduate	2.52	1.33	
		Post-graduate	2.82	1.24	
Gastrointestinal symptoms	Age (in years)	18-20	2.59	1.38	.3941
		21-23	2.74	1.49	
		24-26	2.90	1.64	
		27-29	2.73	1.45	
		>29	2.89	1.27	
	Gender	Male	2.11	0.60	< .0001*
		Female	4.16	0.43	
	Education	Diploma	3.01	1.61	< .0001*
		Graduate	2.43	1.04	
		Post-graduate	2.68	1.42	

For depression, significant differences were also observed for gender and education but not age. Females report higher depression levels (mean = 4.06, variance = 0.39) compared to males (mean = 2.15, variance = 0.89), with a p-value of < .0001. The education level shows significant differences, with diploma holders again experiencing the highest depression (mean = 2.88, variance = 1.76), followed by post-graduates (mean = 2.82, variance = 1.24) and graduates (mean = 2.52, variance = 1.33), with a p-value of .0276.

GI symptoms show significant differences for gender and education, but not for age. Females report higher GI symptoms (mean = 4.16, variance = 0.43) compared to males (mean = 2.11, variance = 0.60), with a p-value of < .0001. Education-wise, diploma holders experience the highest GI symptoms (mean = 3.01, variance = 1.61), followed by post-graduates (mean = 2.68, variance = 1.42) and graduates (mean = 2.43, variance = 1.04), with a p-value of < .0001. These findings underscore the significant impact of gender and education on students' perceptions of anxiety, depression, and Gl symptoms.

The correlation analysis (see Table 6) between anxiety, depression, and GI symptoms among university students reveals strong and statistically significant relationships between these variables. The correlation coefficient between anxiety and depression is 0.674, indicating a strong positive correlation, which suggests that higher levels of anxiety are associated with higher levels of depression among students.

**Table 6:** Correlations between anxiety, depression, and gastrointestinal symptom scales * Statistically significant correlation at p < .05

	Anxiety	Depression	Gastrointestinal symptoms
Anxiety	1		
Depression	0.674*	1	
Gastrointestinal symptoms	0.752*	0.646*	1

The correlation between anxiety and GI symptoms is even stronger, with a coefficient of 0.752. This indicates a very strong positive relationship, suggesting that students experiencing higher levels of anxiety are likely to report more GI symptoms. Similarly, the correlation between depression and GI symptoms is 0.646, also indicating a strong positive relationship. This implies that higher levels of depression are associated with an increase in GI symptoms.

These strong correlations highlight the interrelated nature of psychological and physical health among students. The significant relationships suggest that interventions targeting anxiety and depression may also positively impact GI symptoms, emphasizing the need for comprehensive mental health strategies that address both psychological and somatic aspects of student well-being. The statistically significant correlations (p < .05) affirm the robustness of these findings, reinforcing the importance of addressing these interconnected issues in university health programs.

## Discussion

The findings from this study underscore the significant relationship between anxiety, depression, and GI symptoms among university students, highlighting the complex interplay between mental and physical health within this population. The results align with existing literature, which has identified strong associations between anxiety, depression, and GI symptoms.

Previous studies have documented the prevalence of physical anxiety symptoms such as dryness of mouth, breathing difficulties, and trembling, which were similarly prevalent in our study (Table 2) [17-19]. These symptoms are well-understood as manifestations of the body's stress response and were confirmed to be significant among the students surveyed. A study by Werden [42] found that anxiety disorders were associated with a 2.4-fold increased risk of developing IBS, further emphasizing the strong link between anxiety and GI symptoms.

In terms of depression, our study found that students often reported an inability to experience positive feelings, a lack of initiative, and feelings of worthlessness (Table 3). These findings corroborate previous research indicating that anhedonia, hopelessness, and self-devaluation are core symptoms of depression among university students [25-27]. A recent study [10] found that in a general population sample, a self-reported clinical level of depression was a significant risk factor in predicting GI symptom categories of nausea, heartburn, diarrhea, and constipation, supporting the notion that depression can contribute to GI distress.

GI symptoms, including abdominal pain, frequent diarrhea, and difficulty swallowing, were notably prevalent (Table 4), consistent with earlier studies linking GI distress to mental health conditions [33-35]. A cross-sectional study [10] found that GI symptom severity was positively associated with overall depressive symptom burden and the severity of individual depressive symptoms in young adults seeking outpatient psychiatric care. Our study reinforces the psychosomatic connection, suggesting that anxiety and depression can significantly exacerbate GI symptoms.

The strong correlations observed between anxiety, depression, and GI symptoms (Table 6) highlight the intertwined nature of these conditions. The correlation coefficient of 0.752 between anxiety and GI symptoms, and 0.646 between depression and GI symptoms, indicates that as anxiety and depression increase, so do the reports of GI distress. This supports the notion of a bidirectional relationship, where psychological distress can lead to physical symptoms, and vice versa. A recent review [43] proposed that the gut-brain axis, involving the bidirectional communication between the GI tract and the central nervous system, plays a crucial role in the development and maintenance of both GI and mental health symptoms.

This study also revealed significant differences in anxiety, depression, and GI symptoms based on gender and education level (Table 5). Female students reported higher levels of anxiety, depression, and GI symptoms compared to their male counterparts. This finding is consistent with previous research indicating that women are more likely to experience higher levels of psychological and somatic distress [24,31]. For instance, in a study [42], it was observed that female gender was associated with a significant risk of developing IBS in patients with anxiety disorders, highlighting the gender-specific vulnerability to comorbid GI and mental health issues. The educational level also played a role, with diploma holders reporting the highest levels of anxiety, depression, and GI symptoms. This may be attributed to the additional pressures and uncertainties faced by students pursuing vocational and technical education, compared to those in more advanced academic tracks [20,22].

The ANOVA results showed no significant differences in anxiety, depression, and GI symptoms by age group. This finding suggests that the pressures and stressors affecting mental and physical health are pervasive across different age groups within the university student population, possibly due to the shared experience of academic and social challenges. However, a study [42] found that older age was associated with a higher risk of developing IBS in patients with anxiety disorders, indicating that age-related factors may play a role in the manifestation of comorbid conditions in some populations.

In conclusion, the updated discussion incorporates evidence from recent and similar research studies to strengthen the arguments presented in the original manuscript. By including studies that specifically examine the relationship between anxiety, depression, and GI symptoms, as well as the influence of gender and socioeconomic factors, the discussion provides a more comprehensive and up-to-date understanding of the topic.

Implications

The theoretical implications of this study suggest that the intricate relationship between anxiety, depression, and GI symptoms among university students is deeply interconnected, supporting the psychosomatic model that mental and physical health issues influence each other reciprocally. This underscores the necessity of viewing mental health conditions not in isolation but as part of a broader biopsychosocial framework. Practically, the findings advocate for the implementation of integrated health interventions within university settings. Such interventions should include comprehensive mental health support that addresses both psychological and physical symptoms. Gender-specific and education-level-specific programs could enhance the effectiveness of these interventions, considering the unique stressors faced by different student demographics. By adopting a holistic approach to student health, universities can better support their students' overall well-being and academic performance, potentially reducing the incidence and impact of anxiety, depression, and GI symptoms.

Limitations

This study has a few limitations that should be acknowledged. First, the use of a cross-sectional survey design limits the ability to infer causality between anxiety, depression, and GI symptoms. Second, the reliance on self-reported data may introduce response bias, as students might underreport or overreport their symptoms due to social desirability or recall biases. Third, the study sample, although diverse, was limited to students from three public medical universities in Saudi Arabia, which may affect the generalizability of the findings to students in other regions or academic disciplines. Lastly, potential confounding variables such as lifestyle factors, pre-existing medical conditions, and stressors outside the academic environment were not controlled for, which could influence the observed relationships. Future research should consider longitudinal designs and broader, more diverse samples to validate and extend these findings.

## Conclusions

This study provides significant insights into the relationship between anxiety, depression, and GI symptoms among university students. The findings highlight a strong, positive correlation between these mental health conditions and physical symptoms, emphasizing the intertwined nature of psychological and somatic health. Gender and educational level were identified as important factors influencing the prevalence and severity of these conditions, with female students and diploma holders reporting higher levels of anxiety, depression, and GI symptoms. These results underscore the importance of adopting a holistic approach to student health, integrating mental and physical health interventions to address the complex needs of this population. By implementing comprehensive, tailored health programs, universities can create a supportive environment that promotes the overall well-being and academic success of their students. Further research with longitudinal and diverse samples is needed to deepen our understanding and develop more effective strategies for managing these interrelated health issues.
